# Experiences of eating disorder services for people caring for a loved one with an eating disorder in the UK: national survey

**DOI:** 10.1192/bjo.2024.812

**Published:** 2025-01-17

**Authors:** Hannah Cribben, Rachel Batchelor, Pamela Macdonald, Janet Treasure, Erica Cini, Dasha Nicholls, Carol Kan

**Affiliations:** Centre for Research in Eating and Weight Disorders, Institute of Psychiatry, Psychology & Neuroscience, King's College London, UK; Oxford Institute of Clinical Psychology Training and Research, University of Oxford, UK; East London Eating Disorder Service for Children and Young People, East London NHS Foundation Trust, UK; Division of Psychiatry, Imperial College London, UK; Centre for Research in Eating and Weight Disorders, Institute of Psychiatry, Psychology & Neuroscience, King's College London, UK; and Vincent Square Eating Disorder Service, Central and North West London NHS Foundation Trust, UK

**Keywords:** Feeding or eating disorders, anorexia nervosa, bulimia nervosa, carers, patients

## Abstract

**Background:**

Research suggests that those caring for a loved one with an eating disorder in the UK report unmet needs and highlight areas for improvement. More research is needed to understand these experiences on a wider, national scale.

**Aims:**

To disseminate a national survey for adults who had experience caring for a loved one with an eating disorder in the UK, informed by the findings of a smaller scale, qualitative study with parents, siblings and partners in the UK.

**Method:**

A cross-sectional web-based survey was disseminated to adults who had experience caring for a loved one with an eating disorder in the UK.

**Results:**

A total of 360 participants completed the survey. Participants described experiences of care received in both children and young people's, and adult services. Those receiving care from children and young people's services generally reported more timely care, greater involvement in care and more confidence managing their loved one's symptoms post-discharge. In both settings, participants identified a number of areas for improvement, including more timely access to care, improved transition processes and discharge planning, and increased involvement in their loved one's care.

**Conclusions:**

This survey captures the experiences of individuals caring for a loved one with an eating disorder in the UK. There are identified discrepancies between experiences of care in children and young people services compared with adult services. Clinical implications and recommendations for improvement are discussed, including improved transition and discharge processes, increased involvement of and/or support for carers themselves, and more timely access to support services for the unwell individual.

It is estimated that 1.25 million people in the UK have an eating disorder,^1^ making them among the most common serious mental disorders. Having support from loved ones/carers plays a crucial role in improving treatment outcomes for individuals with eating disorders.^2^ Carers can help in a variety of ways, such as encouraging help-seeking behaviours, strengthening self-esteem, fostering motivation for recovery and advocating for their loved ones to receive access to high-quality care and evidence-based treatment.^3^ For younger patients, carers (usually parents) play a central role as agents of change during the therapy process. However, the responsibility and challenges that come with caring for a loved one with mental disorders, including eating disorders, can make carers vulnerable to experiencing poorer well-being, as well as feelings of distress, guilt, loneliness, loss, uncertainty and worry.^4,5^ Such adverse impacts have been reported in a range of carers, including parents, siblings and partners.^6,7^ These impacts can have potential negative effects on the recovery of people with eating disorders,^8^ highlighting the need for carers to access and receive support. Recent international research has summarised the experiences of parents in caring for their child with an eating disorder,^9,10^ but we do not have a comprehensive understanding of these experiences specifically in a UK context. Indeed, a recent systematic review examined the experiences of accessing support for individuals and carers of those with an eating disorder, including 20 studies from the UK/Ireland. Their results highlighted a lack of primary papers examining these experiences and understanding discrepancies across the healthcare interface (i.e. the point of interaction between different healthcare systems).^11^ For example, it is possible that there will be discrepancies in the resources available in children and young people (CYP) compared with adult services, because of the injection of funding into CYP services following the NHS Long Term Plan.^12^ The National Institute for Health and Care Excellence^13^ guidelines for eating disorders specifies that all caregivers require support. Moreover, Beat,^14^ the national eating disorder charity, and the Academy for Eating Disorders (AED),^15^ the leading international association for eating disorder professionals, published guidelines for improved carer support. Recommendations included enhanced information, skills training, carer involvement, communication with services, assessment of carers’ own needs and monitoring of their well-being (full recommendations are outlined in Supplementary Appendix 1 available at https://doi.org/10.1192/bjo.2024.812). Such care practices have been shown to lead to positive outcomes for both carers and their loved ones with eating disorders.^16^ However, our focus groups exploring the experiential perspectives of parents, siblings and partners of a loved one with an eating disorder identified that the best-practice standards and healthcare rights published by Beat and the AED are not always being followed across several areas.^2,17^ Particular areas that carers identified as requiring improvement included accessing services, the care their loved ones received, staff training, transitioning between services, feedback mechanisms and the involvement, communication and support they received as carers. Therefore, the aim of this study was to create a national survey, informed by our previous focus group findings,^2,17^ to explore the experiences and needs of those caring for a loved one with an eating disorder in the UK, across healthcare interfaces: (a) in-patient versus out-patient versus community, and (b) adult versus CYP services.

## Method

### Study design and participants

This was a cross-sectional, web-based survey undertaken between 14 July 2020 and 14 November 2020. Inclusion criteria were as follows: (a) adults (aged >18 years), (b) self-reported having a loved one who has experienced care for an eating disorder in the UK and (c) fluent in English. For this survey, loved ones have been classified as a parent, partner, sibling or close friend.

Participants were recruited through social media channels of eating disorder charities in the UK, such as Beat (https://www.beateatingdisorders.org.uk/) and F.E.A.S.T. (https://feast-ed.org/), as well as departmental and academic social media accounts. The web survey was accessed through the secured online Qualtrics system.^18^ Participants were required to read an information sheet and provide informed consent on the first webpage of the survey, before they could navigate to further questions. Completion time was approximately 30 min. Four prizes of a £50 online voucher were offered as incentives for participation. Approval for the study was granted by King's College London Ethics Committee (ethics number: HR-19/20-14803).

### Measures and procedure

Findings from our previous focus groups with parents^2^ and partners and siblings^17^ of people with eating disorders informed the content of the questionnaire. The questionnaire was further co-developed with professionals working in eating disorders and our public and patient involvement (PPI) group. Some authors also had lived experience of caring for a loved one with an eating disorder, and were involved with the design, analyses and write-up of the research. The PPI group consisted of two mothers, one father and one partner of individuals with eating disorders. The group participated in the survey design and testing. Their valuable input ensured that the questions conveyed the intended meanings, and the responses available captured their views.

The web survey gathered demographic and clinical information regarding participants and their loved ones. This included gender, age, location, living arrangements and relationship status between participants and loved ones. Clinical information focused on the loved one with an eating disorder, including eating disorder diagnosis, age at diagnosis and duration of illness. To allow for comparisons between healthcare interfaces, data were collected pertaining to the type of care received (in-patient/out-patient/day care and adult/CYP). Drop-down options for diagnosis deliberately included diagnostic labels from a range of classification tools, including the DSM-IV, DSM-5 and ICD-11,^19,21^ to capture all of the diagnoses that we expected to find.

To address the aim of exploring the experiences and needs of those caring for a loved one with an eating disorder in the UK, information was collected with regards to the following: (a) support received, (b) accessing services, (c) most recent experience of care in in-patient/day care/out-patient setting, (d) staff training, (e) transitions and (f) recommendations for service improvement (see Table 1). To address the aim of enabling comparisons between healthcare interfaces, participants were asked to indicate the setting in which their most recent experience of care was provided, and, where applicable, to reflect specifically on their experiences within this setting when answering questions. A full copy of the survey is available in Supplementary Appendix 1. Data were predominantly quantitative in nature, including Likert rating scales and percentage ratings out of 100. Quantitative data were supplemented by open-text feedback.

**Table 1:** Detailed information regarding survey data collected

Data collected	Information
Support received	This section focused on participants’ perceptions of the support provided specifically for them as a carer. Participants responded yes/no/unsure to indicate whether they felt supported in their role, alongside tick box options to indicate the support that they had received, and the support they would have liked to receive.
Accessing services	This section focused on experiences within the first assessment for an eating disorder. Information was gathered using drop-down options regarding knowledge of services, involvement in loved one's assessment, length of time to access assessment, the developed care plan and what support was offered to carers at this time.
Most recent experience of care	This section asked participants to reflect on their loved ones’ most recent experience of care. To allow for comparisons across the interface, carers were asked to indicate whether the care setting was in-patient/out-patient/day care and adult/CYP. Participants answered questions using drop-down options regarding the waiting list times, involvement in their loved one's care, information sharing and treatment/discharge planning. Participants were asked to provide ratings from 0 to 100 regarding communication, involvement in care and information provided about eating disorders and carer support. An open-text box was offered for participants to record any additional comments.
Staff training	The staff training section was organised into two subsections: (a) general practice and (b) eating disorder team. In section (a), participants were asked to rate their confidence (0–100) in professionals to spot the signs for an eating disorder, and refer into appropriate services. In section (b), participants were asked to rate their confidence (0–100) in professionals to treat their loved one and work well together. They were also asked whether they experienced conflicting advice, and whether staff were able to provide the required information or support.
Transition	Participants were asked whether their loved one had ever (a) transitioned from CYP to adult services and (b) transitioned between different eating disorder teams. Participants were asked yes/no/do not know questions regarding the provision of a transition meeting, an allocated professional, a transition summary, gaps in the transition process and the coordination of the transition process. Participants were asked to rate their experience of transition from 0 to 100.
Recommendations	Participants were asked to focus on their most recent experience with an eating disorder team. They responded to yes/no/do not know questions regarding whether feedback and complaint processes were made clear to carers. They were asked to choose their three most important recommendations from a list of 11 recommendations and an open-text option. They were also offered an open-text box for any additional comments.

CYP, children and young persons.

### Statistical analysis

For quantitative data, findings were reported as descriptive statistics, using frequencies and percentages for categorical variables and means and s.d. for continuous variables. Analysis was completed with R software (version 3.6.3 for MacOS, R Core Team, Vienna, Austria; https://www.r-project.org/).^22^ For free-text questions, responses were summarised to supplement quantitative information.

## Results

### Background

Three hundred and sixty participants completed the survey, although some participants did not complete all demographic questions. The majority self-identified as female (*n* = 325; 92.6%) and from England (*n* = 297; 84.6%), with a mean age of 48.7 years (s.d. = 10.0). Most (*n* = 303; 85%) were parents/step-parents, and the majority lived in the same household as their loved one with an eating disorder either all of the time (*n* = 238; 67.8%) or sometimes (*n* = 52; 14.8%). The most common eating disorder was anorexia nervosa. The mean age of a loved one when first experienced eating disorder symptoms was 14.8 years (s.d. = 5.0). Full demographic information is outlined in Table 2.

**Table 2:** Data relating to participants and their loved one with an eating disorder (*N* = 360)

Demographic	*n* (%)
Gender of participant	
Female	325 (92.6)
Male	24 (6.8)
Other	2 (0.6)
Mean age of participant, years (s.d.)	48.7 (10.0)
Relationship of participant to loved one with an eating disorder	
Parent or step-parent	303 (84.2)
Sibling or step-sibling	21 (6.0)
Partner	15 (4.3)
Close friend	9 (2.6)
Does the participant live with their loved one with an eating disorder?	
Yes, all the time	238 (67.8)
Yes, sometimes	52 (14.8)
No	61 (17.4)
Diagnosis of the loved one with an eating disorder	
Anorexia nervosa	285 (79.2)
Atypical anorexia nervosa	20 (5.6)
Bulimia nervosa	12 (3.3)
Eating disorder not otherwise specified	11 (3.1)
Avoidant/restrictive food intake disorder	6 (1.7)
Binge eating disorder	4 (1.1)
Other specified feeding or eating disorder	1 (0.3)
Other eating disorder diagnoses	10 (2.8)
Mean age at onset for the loved one with an eating disorder, years (s.d.)	14.8 (5.0)
Mean length of illness in years for the loved one with an eating disorder, years (s.d.)	4.7 (5.0)
Psychiatric comorbidities for the loved one with an eating disorder	
Yes, concurrently	166 (47.3)
Yes, in the past	28 (7.9)
No	142 (40.6)
Do not know	15 (4.3)
Medical comorbidities for the loved one with an eating disorder	
Yes, concurrently	96 (27.7)
Yes, in the past	20 (5.8)
No	214 (61.7)
Do not know	17 (4.9)

Some participants did not complete all demographic questions.

In terms of services accessed, the most common was CYP out-patient (*n* = 244), followed by CYP in-patient (*n* = 131), adult out-patient (*n* = 123), adult in-patient (*n* = 85), CYP day care (*n* = 44) and adult day care (*n* = 37). Both CYP and adult out-patient services were accessed most frequently, with the mean number of times accessed being 4.2 (s.d. = 1.9) and 3.9 (s.d. = 1.8), respectively.

### Initial assessment

Focusing on the initial assessment, 47.8% of the participants knew how to access services (*n* = 150), and 40.4% of participants felt that their loved one was assessed as soon as necessary (*n* = 127). In addition, 71.7% of participants felt involved in their loved one's first assessment for an eating disorder (*n* = 238). Of those who did not feel involved, 78.3% would like to have been involved (*n* = 94). Further, 37.0% of participants were informed of the contents of their care plan (*n* = 122) and just over 20% felt that an appropriate level of information/support was provided (*n* = 70).

### Experience of care

**Fig. 1:**
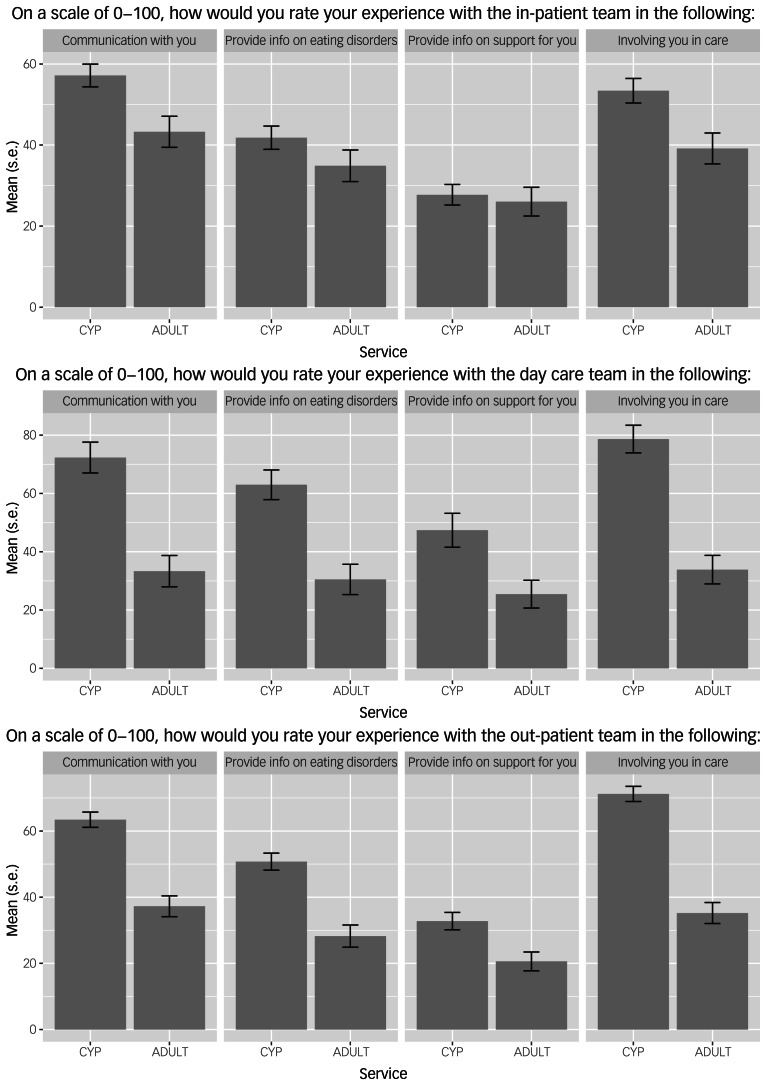
Participants’ rating of overall experience of care in (a) in-patient, (b) day care and (c) out-patient settings, presented as mean (s.d.), with 100% being excellent. CYP, children and young persons.

The experience of care between CYP and adult services was compared across the three settings. The overall experience of care in in-patient, day care and out-patient settings is summarised in Table 3 and Fig. 1.

**Table 3:** Participant ratings of overall experience of care in in-patient, day care and out-patient settings, presented as mean (s.d.), with 0 being terrible and 100 being excellent

	In-patient		Day care		Out-patient	
	CYP	Adult	CYP	Adult	CYP	Adult
Communication with you	57.2 (28.3)	43.3 (31.9)	72.3 (26.4)	33.3 (30.9)	63.4 (29.0)	37.3 (32.4)
Providing you with information about eating disorders	41.8 (2.9)	34.9 (3.9)	63.0 (25.5)	30.5 (30.2)	50.7 (32.0)	28.2 (32.1)
Providing you with information about support for you	41.8 (27.7)	26.0 (28.7)	47.4 (27.3)	25.5 (27.3)	32.8 (32.0)	20.6 (28.0)
Involving you in your loved one's care	53.4 (30.8)	39.2 (31.7)	78.6 (23.7)	33.8 (28.0)	71.2 (29.1)	35.2 (32.2)

CYP, children and young persons.

#### In-patient care

Of those responding to questions regarding in-patient care, 57.8% received care from CYP (*n* = 108) and 42.2% from adult (*n* = 79) services. Some participants did not respond to all questions and therefore the total number of responses for some of the following themes is smaller than *n* = 108 and *n* = 79 for CYP and adults, respectively. Of those who received support in CYP services, 38.9% felt that their loved ones were admitted as soon as necessary, compared with 28.6% in adults (CYP *n* = 42; adult *n* *=* 22); 28.7% in CYP and 35.1% in adult services felt that their loved ones should have been admitted much sooner (CYP *n* = 31; adult *n* = 27). Nearly 70% of respondents in CYP services and nearly 50% of respondents in adult services reported feeling involved in the care of their loved one on admission (CYP *n* = 74; adult *n* = 39). Of those who were not involved, 87.9% of CYP and 74.4% of adult services would have liked to be involved (CYP *n* = 29; adult *n* = 29).

In CYP, 27.1% of respondents and 22.0% in adult services found that the information provided was either extremely or very useful (CYP *n* = 23; adult *n* = 13). Furthermore, 71.8% of respondents in CYP and 55.9% in adult services felt they could talk to staff about the care received by their loved one (CYP *n* = 61; adult *n* = 33). Before discharge, 42.1% of respondents from CYP and 19.7% from adult services felt confident in how to manage their loved ones’ eating disorder (CYP *n* = 35; adult *n* = 13).

Regarding discharge planning, 56.4 and 40.0% of respondents felt involved in discharge planning for their loved ones in CYP and adult services, respectively (CYP *n* = 48; adult *n* = 26). The needs of the family and/or home situation and future health/social care services involvement were felt to be considered in 56.0 and 65.5% of discharge meetings in CYP services (CYP *n* = 47; adult *n* = 55), and 46.1 and 52.3% of discharge meetings in adult services (CYP *n* = 30; adult *n* = 34), respectively. A total of 83.5% of loved ones in CYP and 72.4% in adult services were aware of the discharge plan (CYP *n* = 66; adult *n* = 42). Over 50% in CYP and 37.9% in adult services reported that the discharge plan was comprehensive (CYP *n* = 43; adult *n* = 25), and 71.4% in CYP and 45.5% in adult services knew who to contact if they were concerned about their loved ones (CYP *n* = 60; adult *n* = 30).

#### Day care

For those receiving care from day care settings, 41.1% received care from CYP services (*n* = 28) and 58.9% received care from adult services (*n* = 40). Some participants did not respond to all questions and therefore the total number of responses for some of the following themes is smaller than *n* = 28 and *n* = 40 for CYP and adults, respectively. Of those who received support in CYP services, 53.6% felt that their loved ones were assessed for day care as soon as necessary, compared with 35.9% in adult services (CYP *n* = 15; adult *n* = 14). However, 29.4% in CYP and 30.8% in adult services felt that their loved ones should have received care much sooner (CYP *n* = 5; adult *n* = 12). Over 80% of respondents in CYP services and 33% of respondents in adult services reported feeling involved in the care of their loved one on admission (CYP *n* = 22; adult *n* = 13). Of those who did not feel involved, 80.0% in CYP and nearly 60% in adult services would have liked to have been involved (CYP *n* = 4; adult *n* = 16).

A total of 57% of respondents in CYP services found that the information provided was either extremely or very useful, compared with 13.6% in adult services (CYP *n* = 12; adult *n* = 3). Over 80% of respondents in CYP services and 34.2% of respondents in adult services felt they could talk to staff about the care received by their loved one (CYP *n* = 17; adult *n* = 12). Before discharge, 42.9% of respondents from CYP and 20.6% from adult services felt confident in managing their loved ones’ eating disorder (CYP *n* = 9; adult *n* = 7).

Regarding discharge planning, 52.4% of respondents in CYP services and 20.6% of respondents in adult services felt involved in discharge planning for their loved one (CYP *n* = 11; adult *n* = 7). The needs of the indiviudal were considered with respect to (i) family and/or home situation and (ii) future health/social care services involvement in (i) 57.1% and (ii) 57.1% of discharge meetings in CYP services (family/home *n* = 12; health/social care *n* = 12) and (i) 31.4% and (ii) 28.6% in adult services (family/home *n* = 11; health/social care *n* = 10), respectively. In addition, 47.6% of loved ones in CYP and 38.2% in adult services were aware of their discharge plan (CYP *n* = 10; adult *n* = 13). Discharge planning was noted as comprehensive for 28.6% of respondents in CYP and 20.6% in adult services (CYP *n* = 6; adult *n* = 7). Furthermore, 66.7% of respondents in CYP and 34.2% in adult services knew who to contact if they were concerned about their loved ones (CYP *n* = 14; adult *n* = 12).

#### Out-patient care

For those receiving out-patient care, 58.1% were within CYP (*n* = 173) and 41.9% were in adult (*n* = 125) settings. Some participants did not respond to all questions and therefore the total number of responses for some of the following themes is smaller than *n* = 173 and *n* = 125 for CYP and adults, respectively. Regarding assessment, 57.2% of respondents in CYP and 37.6% in adult services felt that their loved ones were assessed for out-patient care as soon as necessary (CYP *n* = 99; adult *n* = 47). A total of 18.5% in CYP and over 30% in adult services felt that their loved ones should have received care much sooner (CYP *n* = 32; adult *n* = 38). In addition, 90.2% of respondents in CYP and 36.8% of adults reported being involved in the care of their loved one when they started treatment (CYP *n* = 156; adult *n* = 46). For those who were not involved, over 80% of CYP and over 70% of adult services would have liked to have been involved (CYP *n* = 14; adult *n* = 55). Reasons for not being able to be involved included confidentiality issues (particularly adult services not involving families, as their loved one was over 18 years of age), staff shortages and/or high staff turnover resulting in family support not being offered/available, services not acknowledging the impact on families/carers and geographical constraints (family living far away from where their loved one is accessing treatment).

For information sharing, 47.1 and 22.6% of respondents received the right amount of information about their loved ones’ condition/treatment from healthcare professionals in CYP and adult services, respectively (CYP *n* = 81; adult *n* = 28). The type of information provided to carers was mostly with regards to meal plans, how to manage eating disorders and the causes of eating disorders. The most common medium of providing this information was via printed information (for example, a leaflet). A total of 33.8% of respondents in CYP services found that the information provided was either extremely or very useful, compared with 21.4% in adult services (CYP *n* = 52; adult *n* = 12).

Regarding communication, 66.7 and 42.8% of respondents felt they could talk to staff about the care received by their loved one in CYP and adult services, respectively (CYP *n* = 42; adult *n* = 12). Before discharge, 50.8% respondents from CYP services and 25% from adult services felt confident in managing their loved ones’ eating disorder (CYP *n* = 32; adult *n* = 7).

Regarding discharge planning, 47.6% of respondents from CYP and 25% from adult services felt involved in discharge planning for their loved ones (CYP *n* = 30; adult *n* = 7). The needs of the family and/or home situation and future health/social care services involvement were considered in 50 and 43.5% of discharge meetings in CYP services (family/home *n* = 31; health/social care *n* = 27), and 25.9 and 44.4% of discharge meetings in adults services (family/home *n* = 7; health/social care *n* = 12). A total of 27.6% of loved ones in CYP and 24.0% in adult services were aware of the discharge plan (CYP *n* = 16; adult *n* = 6). Further, 32.3% of respondents in CYP and 33.3% in adult services reported that the discharge plan was comprehensive (CYP *n* = 20; adult *n* = 9). Finally, 51.7% of respondents in CYP services and 37.0% of respondents in adult services knew who to contact if they were concerned about their loved ones (CYP *n* = 31; adult *n* = 10).

### Staff training

When asked on a sliding scale from 0 (not at all) to 100 (extremely), moderate confidence was expressed in general practitioners’ ability to spot the signs for an eating disorder (mean 47.7, s.d. 35.4) and refer to the appropriate mental health service (mean 55.8, s.d. 35.7). Similar levels of confidence in the eating disorder team were expressed in their ability to treat patients with an eating disorder (mean 58.3, s.d. 30.8) and work well together (mean 52.2, s.d. 32.8). A total of 61.4% of respondents reported receiving conflicting advice from professionals that affected patient care (*n* = 209). Over three-quarters of respondents felt that staff were available to provide information/support when required (*n* = 260).

### Transition and feedback mechanism

When asked to reflect on transition, 18.3% of respondents indicated that they had been involved in the process of transition from CYP to adult services for eating disorder treatment (*n* = 66). Additionally, 57.6% had a handover meeting set up between the respondents/family members, loved one with an eating disorder and CYP and adult teams (*n* = 38). Further, 51.7% had a key professional identified before their transition to the adult service, where the sole responsibility of the key professional was to work to support the transition for the individual with an eating disorder (*n* = 31).

In addition to transition from CYP to adult services, a substantial portion of respondents reported having experience of their loved ones being transferred between eating disorders teams (44.8%; *n* = 146). Some participants did not respond to all questions and therefore the total number of responses for some of the following themes is smaller than *n* = 146. A total of 71.4% reported that a transfer summary was provided to the adult/new service (*n* = 75). Considering quality of transfer summary, 75.3% rated the transfer summary as adequate (*n* = 55); 39.1% described the transfer as well-coordinated between services, enabling respondents to feel supported (*n* = 52); and 67.9% identified gaps/inefficiencies in the transition process (*n* = 76). This included poor communication between the transitioning teams, both with each other and with families; inaccurate transition summaries; long delays in the transition process; a lack of appropriate adult services to transition to; key professionals not being involved in transition meetings; poor forward planning of the transition process meaning that it felt rushed and disorganised when the patient turned 18 years old; and families not having a named professional to contact with queries during the transition process. The median overall experience of transition was 25 (interquartile range: 5.5–57.5), with 100 being excellent.

Regarding feedback mechanisms, nearly 30% of respondents were asked by the eating disorder team to give their views on the quality of care received by their loved one, such as through conversation or a questionnaire (*n* = 93). Nearly 25% saw or were given information explaining how to complain about the care received (*n* = 73). Only 34.6% were aware of how their feedback would be acted on by the eating disorder service (*n* = 18).

### Recommendations

The overall care received by the loved one was rated as 54.8 (s.d. 28.3), with 100 being excellent. This indicates that the care providers should consider improvements specifically with regards to the experiences of those caring for a loved one with an eating disorder who accesses services. When asked to select the three most important recommendations for services, carers most frequently selected (a) ‘removing criteria for referral to outpatient eating disorder unit for an assessment (e.g. body mass index cut-off)’, (b) ‘organising a joint meeting with you, your loved one with an eating disorder and a professional during treatment’ and (c) ‘offering a skills-based training for supporting loved ones’. There were also high endorsements of the recommendation to ‘equip primary care teams to diagnose eating disorders’.

## Discussion

This study aimed to explore the experiences and needs of those caring for a loved one with an eating disorder across the UK, using an online survey. This survey was informed by findings from our previous focus group studies with carers.^2,17^ The participants described care received in both CYP and adult services, across all settings. In general, participants with a loved one receiving care from CYP services reported more timely care, greater involvement during treatment and more confidence in managing eating disorders post-discharge, although our results highlight a need for improvement across all settings.

### Findings and clinical implications

Timely access to eating disorder services was an area of concern raised by many respondents. Just over half of participants knew how to access services for initial assessment, in the context of significant delays in receiving appropriate treatment. For example, only 38.9% of loved ones in CYP and 28.6% in adult services reported that their loved ones were admitted as soon as necessary. This is concerning, given that prompt treatment can reduce chronicity and enhance recovery.^23^ In addition, loved ones expressed moderate confidence in the ability of general practitioners (GPs) to spot the signs of an eating disorder and refer to the appropriate mental health service.

In the UK, GPs act as an important gateway for patients to access care for eating disorders.^24^ Despite this, recent qualitative research with GPs practicing in the UK suggests that these professionals feel undereducated, underresourced and underequipped to respond to concerns of patients with eating disorders in primary care.^25^ This further highlights the need for improved training and enhanced professional resources for GPs in the UK. Encouragingly, all CYP services in England received significant government funding to partake in a large-scale transformation programme to support the development of dedicated community eating disorder services.^26^ This initiative included a newly developed training curriculum to upskill the workforce. Importantly, requirements for these new services stipulated that they should be accessible via self-referral, thus enabling more timely access to specialist support, with guidelines indicating 1 week for urgent referrals and 4 weeks for more routine referrals. However, it is worth noting that progress toward these targets was affected by the surge in referrals to CYP community eating disorder services during the COVID-19 pandemic,^27^ indicating the urgent need to ensure adequate service provision in CYP settings to meet clinical demands. The need for dedicated and extensive training in eating disorders for professionals has been recognised by NHS England; the organisation has commissioned whole-team training for both CYP^26^ and adult eating disorder services.^28^ This is an important step toward upskilling professionals, and is an ongoing process.

The transition process between CYP and adult services, and between eating disorder teams, could be significantly improved; the overall experience was rated poor (mean: 35.5, with 100 being excellent). Although a transfer/handover meeting or a transfer summary were often provided, only 54.4% had a key professional identified before transition to the adult service. Indeed, mirroring the findings in this survey, a recent systematic review examining problematic age transitions in eating disorders highlighted a number of issues, including a lack of involvement and information for loved ones during the transition process, poor communication and differing treatment approaches between CYP and adult services.^29^ Indeed, this finding is not unique to those caring for a loved one with an eating disorder, and has been reported by caregivers experiencing a transition in care for their loved ones with psychosis, bipolar disorder and depression.^30^ It is important that services involved in transition address the reported gaps in this process by (a) ensuring that loved ones are involved with, and aware of, the process; (b) ensuring that correct information is passed to the patient, their loved ones and the service they are transitioning to; (c) an appropriate care plan is in place and has been communicated with the service they are transitioning to and the family, with sufficient time before the transition takes place; and (d) identifying the most suitable service for patients with more unique needs that may not be adequately met in ‘traditional’ eating disorder services (e.g. avoidant/restrictive food intake disorder). For professionals seeking further pragmatic guidelines for managing transitions in eating disorder teams, the relevant national guidelines should be consulted.^31,32^

Another potential area for improvement is the involvement of loved ones in care – this includes improved information sharing, increased involvement in treatment and support for loved ones themselves. Just over 20% of respondents indicated that an appropriate level of information/support was provided during assessment. Indeed, this finding appears to be consistent with the broader experiences of carers within the NHS, including those whose loved ones are accessing care in in-patient settings^33^ and secondary mental healthcare teams.^34^ It is important to equip loved ones with information and support to manage eating disorders when they first access services, especially given the current long waits for NHS mental health treatment.^35^ Indeed, key recommendations for early intervention in eating disorder pathways mention the routine involvement of loved ones, including the provision of carer support.^36^ For example, parent group models for the early intervention of young people with eating disorders are associated with improved knowledge and confidence for parents, and improved eating disorder psychopathology and weight gain for young people.^37,38^ The success of group models extends beyond just parenting groups, with positive outcomes reported for a group attended by partners, siblings and parents caring for CYP and adults with an eating disorder.^39^ We can also learn from literature in other severe and enduring mental health conditions, such as psychosis, where UK-based research has reported improvements in measures of carer distress and/or well-being following interventions such as carer education and information groups,^40,41^ and specialist psychological support for carers.^42^

There is a marked discrepancy in the proportion of loved ones who report feeling involved in care between CYP (range: 69.2–90.2%) and adult (range: 33.3–49.4%) services. In addition, loved ones who received day care and out-patient adult services reported poorer communication from staff and lack of information about eating disorders being provided, compared with those receiving in-patient treatment. This finding is not unique to the eating disorder carer population; research on carers of loved ones in secure mental health facilities also report challenges with communication, and staff working in these facilities report difficulties negotiating the complexities of information sharing and data confidentiality.^43^ It is clear that increased emphasis on establishing clear communication channels between eating disorder services and loved ones is needed, provided that patients have consented to this. If appropriate consent cannot be obtained, avenues need to be explored regarding how loved ones can contact professionals if they have concerns about their loved ones, without violating patient confidentiality. Indeed, we may be able to learn from an NHS England toolkit for carer support and involvement in secure mental health services, which outlines specific guidelines for balancing involvement and confidentiality when working with carers.^43^

More resources need to be dedicated to discharge planning across all settings, given that under half of respondents in CYP services (42.1–50.0%), and under a quarter in adult services (19.7–25.0%), felt confident in managing their loved ones’ eating disorders on discharge. Indeed, a recent qualitative study on patients being discharged from intensive hospital treatment for anorexia nervosa in the UK highlighted significant barriers to continued recovery, including poor discharge planning and an insufficient support system in the community.^44^ It is clear that involving loved ones in discharge planning, providing more comprehensive discharge plans and ensuring that loved ones are aware of who to contact if they were concerned will be useful starting points to improve these experiences. Furthermore, the sustained involvement and upskilling of loved ones throughout high-intensity treatment programmes would likely improve loved ones’ confidence, and patients’ perceived support, in the management of the eating disorder post-treatment and in community settings.

In summary, our findings highlight the importance of services prioritising collaborative working and effective communication throughout the patient journey, from receipt of referral to discharge, and particularly when transitioning between services. To allow for improved access to services, we would recommend that services keep an open mind on the diversity of eating disorder presentations, avoiding a stereotyped view of what constitutes an eating disorder presentation. We would also recommend that services consider how they are supporting the patient's journey within the service, being mindful of the importance of continuity of care as well as ensuring that a case-load is regularly reviewed to ensure that timely discharges are enacted, ensuring resource optimisation. Finally, we recommend that loved ones are invited to be involved at all stages of the patient journey, while still respecting patient's wishes and confidentiality. Loved ones include the individual's main carer(s) and wider systemic networks, such as siblings, partners and extended friends/families. These individuals can be offered skills-based training and practical support to enable carers to feel more in control of the care for their loved one, and to ensure a robust relapse prevention plan.

### Limitations

The findings of the current study need to be viewed in the context of several limitations. Although our study included a large number of carers with experiences of a range of therapeutic settings, most participants were female parents/step-parents of a loved one with anorexia nervosa. Participants were also mostly White British, with other ethnic backgrounds being comparatively underrepresented. The mean duration of illness was 4.7 years, therefore the survey may capture experiences of care for individuals who have a longer duration of illness, whereas many CYP and young adults may have a much shorter duration of illness (e.g. those accessing first episode rapid early intervention for eating disorders (FREED) services).^45^ It is also possible that respondents in our study reflected those with negative service experiences, who were more motivated for change, potentially biasing our findings. It would be beneficial for future research to seek to recruit a more diverse sample by expanding recruitment channels, such as by advertising the research in FREED services, to capture experiences of those with a much shorter duration of illness.

In making comparisons between experiences of care in CYP and adult settings, it is important to consider that carers are routinely involved in CYP care unless a capacious young person aged ≥16 years requests that they are not involved; unfortunately, we did not have the data to establish whether this was the case for carers who reported not being involved in care. Furthermore, our comparisons did not allow for consideration of the likely difference in domestic circumstances between those accessing care in CYP and adult services (i.e. that those accessing care in CYP services are more likely to still live with their parent/carer). Future research should seek to address this by drawing direct comparisons between the perspectives of carers who share a home with their loved one versus those who live independently.

Given that our survey was informed by our previous focus group findings, and co-developed with our PPI group, we did not include a direct, validated measure of carer distress. This is an outcome that is frequently reported in carer research,^8,46^ and it would be beneficial for future research to compare carer distress across the healthcare interface. It would also be interesting to compare the reported standard of carer support dependent on loved one's clinical diagnostic factors, including comparisons of carer perspectives for differing illness durations, eating disorder diagnosis and age at onset. This would help us to identify particular populations in which carer support may require particular attention for improvement, although we acknowledge that published national guidelines should be met by all care providers regardless of eating disorder diagnostic factors.

Furthermore, there was a significant level of missing data throughout the survey, as responses were optional, not mandatory. It was not possible to assess the reasons for missing data; it might be that participants chose not to respond to questions that were not relevant to their experiences, or it might be that they did not respond to questions that were relevant, but too upsetting/distressing to reflect on and respond to.

The survey was conducted online, and those without access to the internet or appropriate devices may have been unable to participate. Their perspectives may have added great value, especially given the changes in care delivery in 2020 as a result of the COVID-19 pandemic. Thus, future research should seek to recruit a more diverse sample and utilise accessible data collection methods to increase generalisability. The survey is retrospective, and there have been various initiatives/changes to eating disorder service provisions in the UK.

In conclusion, this survey captures the experiences of individuals caring for a loved one with an eating disorder in the UK. Although our findings indicate a key discrepancy between the experiences of those accessing support in CYP versus adult services, the data suggests that there are gaps in the provision of carer support across all settings. There is, therefore, a need for both CYP and adult services to consider the key areas identified for improvement, including more timely access to support services, improved transition processes and discharge planning, and increased involvement of and/or support for carers themselves at all stages of their loved ones’ treatment journey.

## Supporting information

Cribben et al. supplementary material Cribben et al. supplementary material

## Data Availability

The data that support the findings of this study are available on reasonable request from the corresponding author, H.C. The data are not publicly available due to containing information that could compromise the privacy of research participants.
