# Supplementary material for: Experiences of eating disorder services for people caring for a loved one with an eating disorder in the UK: national survey
Source: BJPsych Open. 2025 Jan 17;11(1):e17. doi: 10.1192/bjo.2024.812 (PMC11795176; doi:10.1192/bjo.2024.812)
Supplement: Cribben et al. supplementary material [file S2056472424008123sup001.docx]

**Appendix 1: Published Guidelines for Improving Carer Support**

**Academy for Eating Disorders**

1. Access to Quality Care

All patients have the right to immediate care for medical and/or psychiatric instability, followed by timely and non-discriminatory access to appropriate specialty care.

1. Respect

All patients, caregivers, and family members have the right to be treated with respect throughout the assessment, planning and treatment process. Patients and carers should never be judged or stigmatized based on symptoms, behaviours or past treatment history.

1. Informed consent

When making healthcare decisions, patients and caregivers have the right to full disclosure by healthcare professionals about treatment best-practices, risks, costs, expected service outcomes, other treatment options, and the training and expertise of their clinicians.

1. Participation

Families and other designated carers have a right to participate in treatment as advocates for the best interests of their loved-ones. Caregiving responsibilities and degrees of participation will necessarily vary depending on the age, mental state and diagnosis of the patient, as well as the caregiver’s skills, availability, personal health, resources and other circumstances.

1. Communication

All patients and carers have the right to establish regular ongoing communications through clearly defined channels. Caregivers and family members have the right to communicate their observations and concerns to professionals and to receive information when the patient’s medical and/or psychiatric safety is threatened or at risk.

1. Privacy

All patients and carers have a right to expect their health professionals to understand, communicate, and respect the applicable privacy or age-of-consent regulations that govern the communication of health and treatment information, as well as the circumstances that may override privacy concerns or transfer authority regarding treatment decisions.

1. Support

All caregivers have a right to receive information, resources and support services to help them understand and carry out the expectations and responsibilities of their roles as partners in treatment.

REFERENCE: Academy for Eating Disorders. World Eating Disorder Healthcare Rights. An AED Global Blueprint for promoting Excellence in Care through Patient-Carer-Professional Partnerships. 2017; Available from: https://higherlogicdownload.s3.amazonaws.com/AEDWEB/27a3b69a-8aae-45b2-a04c-2a078d02145d/UploadedImages/Advocate/World_ED_Rights_Flyer_04_03_2017.pdf

**Beat**

1. Have a policy that ensures optimum involvement of and support for all carers as soon as a loved one starts treatment.
2. Train all service staff in the application of the policy and these standards with particular focus on the importance of carers as a resource for recovery.
3. Provide all carers with useful and comprehensive information about eating disorders when their loved one receives a diagnosis.
4. Offer all carers and siblings an assessment of their own needs when a loved one receives an eating disorder diagnosis, continue to monitor their wellbeing throughout the sufferer’s treatment and, where necessary, refer carers to specialist services.
5. Offer all carers options for peer-to-peer support.
6. Offer all carers opportunities to learn the necessary skills to provide optimum support for their loved ones.
7. Inform and engage carers when a loved one faces a transition between services and ensure that effective communication between both services and carers takes place.
8. Provide a mechanism by which carers’ input and feedback is sought and acted upon.

REFERENCE: Beat. Best Practice in the Engagement and Empowerment of Families and Carers Affected by Eating Disorders. [Internet]. 2019 [cited 2023 Oct 20]. Available from: https://beat.contentfiles.net/media/documents/family-empowerment-guidance-1_fP1wHWr.pdf
